# Role of Corticotropin Releasing Factor in the Neuroimmune Mechanisms of Depression: Examination of Current Pharmaceutical and Herbal Therapies

**DOI:** 10.3389/fncel.2019.00290

**Published:** 2019-07-02

**Authors:** Yizhou Jiang, Tangming Peng, Uma Gaur, Marta Silva, Peter Little, Zhong Chen, Wei Qiu, Yandong Zhang, Wenhua Zheng

**Affiliations:** ^1^Center of Reproduction, Development and Aging and Institute of Translation Medicine, Faculty of Health Sciences, University of Macau, Macau, China; ^2^Department of Biology, Southern University of Science and Technology, Shenzhen, China; ^3^Department of Neurosurgery, The Affiliated Hospital of Southwest Medical University, Luzhou, China; ^4^Neurosurgical Clinical Research Center of Sichuan Province, Luzhou, China; ^5^School of Pharmacy, Pharmacy Australia Centre of Excellence, The University of Queensland, Woolloongabba, QLD, Australia; ^6^Key Laboratory of Medical Neurobiology of the Ministry of Health of China, College of Pharmaceutical Sciences, Institute of Pharmacology and Toxicology, Zhejiang University, Hangzhou, China; ^7^The Third Affiliated Hospital, Sun Yat-sen University, Guangzhou, China

**Keywords:** depression, neuroimmune system, corticotropin releasing factor, HPA axis, stress

## Abstract

Approximately 3% of the world population suffers from depression, which is one of the most common form of mental disorder. Recent findings suggest that an interaction between the nervous system and immune system might be behind the pathophysiology of various neurological and psychiatric disorders, including depression. Neuropeptides have been shown to play a major role in mediating response to stress and inducing immune activation or suppression. Corticotropin releasing factor (CRF) is a major regulator of the hypothalamic pituitary adrenal (HPA) axis response. CRF is a stress-related neuropeptide whose dysregulation has been associated with depression. In this review, we summarized the role of CRF in the neuroimmune mechanisms of depression, and the potential therapeutic effects of Chinese herbal medicines (CHM) as well as other agents. Studying the network of CRF and immune responses will help to enhance our understanding of the pathogenesis of depression. Additionally, targeting this important network may aid in developing novel treatments for this debilitating psychiatric disorder.

## Introduction

Depression, also termed as clinical depression or major depressive disorder (MDD), is a common but serious mental disorder affecting the quality of human life. Depression is characterized by discrete episodes of more than 2 weeks’ durations with distinct changes in cognition, and neurovegetative functions and inter-episode remissions (American Psychiatric Association, 2013). Depression is one of the most common mood disorders currently affecting approximately three percent of the world’s population (GBD 2015 Disease and injury incidence and prevalence collaborators, 2016), and is one of the leading contributors to the global burden of diseases. Depression shows gender specificity in which women have a lifetime incidence of depression two times greater than men. Also depression is shown to be associated with elevated risk of cardiovascular, cerebrovascular disease and other forms of disease-related mortality (Steffens et al., 1999; Bradley and Rumsfeld, 2015). In addition, patients with depression have higher suicidal tendency which makes it a potentially life-threatening mental disorder (Duman et al., 2016).

Corticotropin releasing factor (CRF) was originally identified by Vale et al. (1981). CRF is a key regulator of the hypothalamic-pituitary-adrenal (HPA) axis, which is the most important neuroendocrine system mediating the stress response. Upon stress exposure, CRF is released from the hypothalamus and it stimulates the production of a series of down-stream stress hormones, including adrenocorticotropin (ACTH) and glucocorticoids (Belvederi Murri et al., 2014). Glucocorticoids in turn inhibit the endocrine activity of the hypothalamus and pituitary gland, forming a negative feedback loop. This feedback loop is vital for the regulation and homeostasis of the stress response system (Slominski et al., 2013). Dysregulation of the HPA axis has extensive effects on the body, and triggers a series of behavioral, physiological, and metabolic responses (Bao and Swaab, 2010; Swaab et al., 2005). HPA axis hyperactivity is a common finding in the pathology of depression (Kinlein et al., 2015). In depression patients, overproduction of CRF was found in parallel with changes in other components of the HPA axis (Lightman, 2008). Therefore, CRF is believed to contribute to the symptoms of depression by regulating activity of the HPA axis.

The immune system serves as the first line of defense against multiple harmful stimuli from the environment. In mammals, the immune system can be divided into two anatomically distinct components: the neuroimmune system and the peripheral immune system. The peripheral immune system consists of different immune cells mainly derived from multipotent hematopoietic stem cells in the bone marrow, such as lymphocytes, mast cells, phagocytes, macrophages, neutrophils, dendritic cells, and natural killer cells (Hashimoto et al., 2011; Hodes et al., 2015). The primary residential immune cells of the neuroimmune system are glial cells (Gimsa et al., 2013; Beardsley and Hauser, 2014). Disorders of the immune system are associated with several chronic diseases (O’Byrne and Dalgleish, 2001), and interactions between the nervous system and the immune system play an essential role in depression (Wohleb et al., 2016). Previous studies have shown that CRF receptors are widely expressed in T cells and glial cells (Stevens et al., 2003; Chatoo et al., 2018). Also, immune cell dysfunction has been observed in depression and chronic exposure to CRF and glucocorticoids inhibits T-cell proliferation (Oh et al., 2012; Jin et al., 2016). Additionally, the expression levels of glial fibrillary acidic protein (GFAP), a marker of astrocytes, is found to be decreased in patients suffering with depression (Miguel-Hidalgo et al., 2000). Cytokines, including interleukin-6 (IL-6), interleukin-1 beta (IL-1β), tumor necrosis factor alpha (TNFα) and interleukin-10 (IL-10), can induce the secretion of CRF upon exposure to stress, and CRF can in turn mediate the level of these cytokines (Kariagina et al., 2004; Chen H. et al., 2018). Thus, it is suggested that the CRF is a key regulator of immune responses in depression. Novel antidepressants can be developed based on the regulatory role of CRF in depression. For example, a large number of Chinese herbal medicines (CHM) hold potential for treating depression because of their abilities to suppress inflammation and normalize elevated CRF levels. Drugs directly modulate CRF signaling and HPA axis activity, such as CRF1 antagonists, can also be potent antidepressants. This review summarizes the evidence highlighting the role of CRF in the neuroimmune regulation of depression and provides a biological basis for developing effective treatments for this psychiatric disorder.

## Neurobiology of Depression

Depression is a disorder with complex pathogenesis which is not well understood because of highly variable pathophysiological course. Familial studies suggest that depression is a heterogeneous mental disease (Belmaker and Agam, 2008). Besides genetic factors, environmental adversities like overall health status, emotional abuse and social problems are also risk factors that lead to depression (Li et al., 2016). At the moment, there is no established mechanism for the interaction between the genetic and environmental factors involved in the onset and development of the depression (Otte et al., 2016).

The mammalian stress response is a complex biological process driven by interactions between the brain and peripheral systems such as the immune and cardiovascular systems (McEwen, 2007). Preclinical and clinical studies have demonstrated that stress and depression are associated with neuroplasticity which is change in the morphology of neurons, alterations in the connectivity and activation of neural networks in a regionally dependent manner (Duman, 2014). Atrophy and loss of neurons and glial cells are seen in the brains of depressed subjects and a reduced volume of hippocampus and cortical brain regions is observed in the pathogenesis of depression (Otte et al., 2016). Changes in dendritic spine density, dendritic length and branching patterns have been described in the hippocampus, amygdala, and prefrontal cortex in response to stress (Davidson and McEwen, 2012). Besides impaired neuroplasticity, decreased neurogenesis in the dentate gyrus (DG) of the hippocampus has also been found in brain of depressed patients (Samuels and Hen, 2011). Looking at the above-mentioned evidence it can be stated that depression affects an individual by changing the neural structures and networks.

## Corticotropin-Releasing Factor (CRF) and HPA Axis: an Overview

Corticotropin-releasing factor (CRF), also termed as corticotropin releasing hormone (CRH), is a 41-amino acid polypeptide. The CRF family also includes three urocortins apart from CRF which are urocortin 1, urocortin 2, and urocortin 3 (Keck, 2006). Members of the CRF family bind to two type of receptors: Corticotropin-releasing factor receptor 1 (CRF1) and Corticotropin-releasing factor receptor 2 (CRF2) which are expressed differently in the nervous system and peripheral tissues. CRF1 is highly expressed in the brain, cerebellum, and pituitary, with a lower expression in peripheral tissues such as skin and adrenal gland (Potter et al., 1994). The expression of CRF2 in CNS is more limited being restricted primarily to subcortical areas such as the hypothalamus and amygdala (Reul and Holsboer, 2002a). CRF2, however, is widely expressed in peripheral tissues including heart, lung, adrenal gland, ovaries and testes (Naughton et al., 2014; Ketchesin et al., 2017).

Corticotropin releasing factor is a key component of the HPA axis. The HPA axis is composed of the hypothalamus, the pituitary gland and the adrenal glands and is a major regulator of endocrine stress response (Keck, 2006). Different brain regions are involved in the stress response system, such as amygdala, hippocampus and the prefrontal cortex (PFC) (Bao and Swaab, 2010). During the stress state, the neuronal activation in these regions converges on the hypothalamus and activates the endocrine stress response (Waters et al., 2015). Typically, CRF is secreted by the median paraventricular nucleus (PVN) in the hypothalamus (as a response to various stressors) and released from the terminals of secretory neurons. CRF is transported by the local vascular system, and stimulates the pro-opiomelanocortin (POMC) transcription and adrenocorticotropic hormone (ACTH) release (also named corticotropin) by binding to CRF1 in the anterior pituitary gland (Lightman, 2008; Slominski et al., 2013). ACTH acts on the adrenal cortex resulting in the synthesis and release of glucocorticoids (cortisol in humans and corticosterone in rodents), which have broad biological effects in the body (Arborelius et al., 1999; Slominski et al., 2013). Glucocorticoids are the main end effectors of HPA activation and also exert negative feedback effects on the hypothalamus and the pituitary gland to inhibit CRH and ACTH production, respectively. Two types of glucocorticoid receptors have been identified: the mineralocorticoid receptor (MR) and the glucocorticoid receptor (GR). Glucocorticoids act on these two kinds of receptors to terminate the stress response (Bao and Swaab, 2010).

## Dysfunction of CRF and HPA Axis in Depression

The HPA axis mediates the endocrine stress response in both basal and pathological conditions. Hyperactivity of HPA axis has been observed as one of the most fundamental mechanisms in the pathophysiology of psychiatric disorders, including depression (Vreeburg et al., 2009). Increased concentrations of CRF in cerebrospinal fluid and CRF mRNA expression in the PVN have been observed in depression patients (Nemeroff et al., 1984; Raadsheer et al., 1995). ACTH and cortisol levels increase in parallel with the hypersecretion of CRF and result in adrenal hypertrophy (Lightman, 2008; Wang et al., 2017). The hyperactivity of HPA axis is accompanied by an impaired HPA negative feedback, and result in hypercortisolemia (de Kloet et al., 2005). Long-lasting abnormal HPA axis activity disrupts endocrine system homeostasis, resulting in a series of physiological, behavioral and mental consequences, and drives the pathogenesis of psychiatric disorders including depression (Bao et al., 2012).

The role of the HPA axis in depression is age-dependent. HPA axis hyperactivity is a common finding in younger patients (Murphy, 1991; Vreeburg et al., 2009). However, results of studies focusing on older patients are mixed. Consistent with findings in younger adults, high cortisol levels were also found in some older depressed subjects (Gotthardt et al., 1995; O’Brien J. T. et al., 2004). Inversely, decreased serum and urinary cortisol levels were observed in other older patient samples (Morrison et al., 2000; Oldehinkel et al., 2001). These findings suggested that both hyper as well as hypoactivity of the HPA axis are implicated in late-life depression (Ancelin et al., 2017). The hypocortisolemia could be due to the chronic exhaustion of the HPA axis (Bremmer et al., 2007). With increasing age, patients with depression show a greater change on the HPA axis activity compared to people without depression, especially in circulating cortisol and ACTH levels (Stetler and Miller, 2011; Belvederi Murri et al., 2014). the HPA axis gets more and more vulnerable to dysregulation with increasing age (Ancelin et al., 2017). This change may be caused by age-related changes in different elements of the HPA axis, such as increasing instability of MRs and biosynthetic dissociation of adrenocortical secretion (Ferrari et al., 2001; Berardelli et al., 2013).

Interestingly, the prevalence of depression in women is several times greater than that in men (Kessler et al., 1993). Sex differences in CRF receptors have been found in almost all brain regions (Weathington et al., 2014). Given the association between CRF and depression, it has been hypothesized that CRF receptors may mediate the gender–dependent prevalence of depression (Waters et al., 2015). In adult rats, CRF1 binding in females is overall greater than that in males, with higher binding in accumbens (ACC), dorsal CA3 and subregions in basal forebrain such as nucleus accumbens shell (AcbS) and olfactory tubercle (OT) (Weathington et al., 2014). Females also have higher CRF2 binding in lateral septum, whereas in other brain regions, such as posterior bed nucleus of the stria terminalis (BST) and ventromedial hypothalamus, males have greater CRF binding (Weathington et al., 2014; Beery et al., 2016). The sex differences of CRF receptors may be a result of evolutionary change to adapt the different adult social behavior that benefits the reproductive success (Weathington et al., 2014). Similar gender bias has also been observed in the key symptoms of depression such as hyperarousal and inability to concentrate, and this bias has been associated with gender differences in CRF regulation (Bangasser et al., 2016). Therefore, according to the published literatures, gender differences in CRF regulation and symptoms of depression strongly support the involvement of CRF in depression.

The CRF system also has a vital role both in stress responses and depression. In depression, excess glucocorticoid levels, caused by hyperactivity of the HPA axis, result in neuronal damage and immune disturbances (Reul and Holsboer, 2002b; Koutmani et al., 2013). CRF stimulates neurogenesis and attenuates the neuronal damage on neural stem/progenitor cells caused by glucocorticoids in mice (Koutmani et al., 2013). Increased numbers of CRF expressing neurons and elevated CRF mRNA expression were found in the PVN of hypothalamus of patients with depression (Raadsheer et al., 1994, 1995). The dysregulation of CRF caused extensive negative effects on the body, such as reduction in appetite, stress-induced analgesia, sleep disturbances, and anxiety (Swaab et al., 2005; Bao and Swaab, 2010). These effects can be mimicked in experimental animals by intracerebroventricular injection of CRF (Holsboer et al., 1992; Holsboer, 2001). CRF overexpression in CNS of mice caused stress-induced hypersecretion of stress hormones and depression-like behaviors (Lu et al., 2008). CRF acts through CRF1 and CRF2 receptors to regulate the depressive-like behaviors, and these receptors play different roles in stress-induced HPA response. Restraint stress induced a rapid and strong down-regulation of hippocampal CRF1 receptor mRNA, while CRF2 receptor mRNA was upregulated in the same region (Greetfeld et al., 2009). Mice lacking CRF1 receptor showed impaired stress-induced HPA response (Muller et al., 2000). In contrast, CRF2-deficient mice showed increased depression-like behaviors (Bale and Vale, 2003; Todorovic et al., 2009), and this effect may be due to elevated hippocampal CRF1 receptor activity caused by MEK/ERK pathway activation in the absence of CRF2 (Todorovic et al., 2009). CRF1 receptor has an essential role in mediating the effect of CRF on HPA axis. A study in rats proved that chronic forced swim stress-induced depressive-like behaviors required the activation of CRF/CRF1 signaling in the basolateral nucleus of the amygdala (Chen L. et al., 2018). Mice lacking CRF2 receptor showed early termination of HPA response, which indicates that CRF2 receptor may be involved in the maintenance of HPA drive (Coste et al., 2000). CRF receptors are widely expressed in the CNS. Therefore, the CRF driven regulation of stress-coping behaviors can be independent of the HPA axis activity. Decreased anxiety was observed in a mouse model where CRF1 was inactivated in anterior forebrain and limbic brain structure while functioning normally in the pituitary (Muller et al., 2003). Taken together, these findings suggest a homeostatic role for CRF in the nervous system. Dysregulation of CRF may cause a series of stress-related diseases which include depression as well. The above discussed roles of CRF1 and CRF2 receptors toward CRF regulation, which leads to the development of depression, might help in better understanding this stress-related psychiatric disorder. Therefore, normalizing the abnormal CRF secretion or blocking the CRF receptors can be effective strategies for the treatment of depression.

## Neuroimmune System and Depression

The earliest indication demonstrated that depression is likely to be associated with inflammation, as it is reported that patients treated with recombinant human interferon alpha developed psychiatric complications (Renault et al., 1987). Subsequently, immune variations have also been observed in depressed subjects. The degree of neutrophilia, monocytosis, and leukocytosis is positively related with the severity of depression, which indicates that an inflammatory cascade might be linked to depression (Maes et al., 1992). However, mitogen-induced lymphocyte proliferation and natural killer cell activity were found to be inhibited in depression patients (Herbert and Cohen, 1993). Furthermore, elevated serum levels of several pro-inflammatory cytokines, such as TNFα, IL-1β, and IL-6, have also been detected in patients with depression (Howren et al., 2009; Dowlati et al., 2010). Therefore, the possibility of depression resulting from inflammatory processes, cannot be ruled out.

The involvement of the immune system in the pathogenesis of depression is also indicated by high comorbidity rates between depression and other diseases associated with chronic inflammation, such as diabetes, cardiovascular disease and cancer (Evans et al., 2005). The chronic inflammation underlying in these disease states is a possible mediator or driver of the progression of depression (Wohleb et al., 2016). Besides the systemic diseases, psychosocial or environmental stress is another important contributor to depression (Christoffel et al., 2011). A study on C57BL/6 mice demonstrated that social defeat stress can lead to depressive-like behavior (Iniguez et al., 2014). Cytokine profiles for different animal models of depression indicates that various forms of stress exposure induces the release of pro-inflammatory cytokines such as INF-γ, IL-1β, and IL-6 (Hodes et al., 2015), which implicates immune responses as an underlying mechanism of depression caused by stress.

## CRF, Cytokines, and Immune Cells in Depression

The peripheral immune system and the neuroimmune system are two distinct compartments of the immune system. Bidirectional molecular pathways have been described between the peripheral immune system and the neuroimmune system which enable the immune communication (Wohleb et al., 2016). The blood-brain barrier (BBB) mediates the trafficking of peripheral immune cells into the CNS and the exchange of cytokines between the blood and the CNS (Erickson et al., 2012). Cytokines produced in peripheral immune cells, like IL-6 and IL-1β, can act on glial cells and neurons in the CNS (Hodes et al., 2015).

Corticotropin releasing factor and HPA axis activity are known to be modulated by cytokines (Pan et al., 2006). Cytokines and their receptors are expressed in both CNS and PNS (Hopkins and Rothwell, 1995). Lipopolysaccharide (LPS) injection into experimental animals induced the synthesis of peripheral pro-inflammatory cytokines such as IL-1, IL-6, and TNFα. These cytokines can cross the BBB and regulate the activity of the HPA axis through multiple cytokine receptors (Utsuyama and Hirokawa, 2002). Depression is associated with the pro-inflammatory cytokine (IL-1, IL-6, and TNFα) via regulation of CRF (O’Brien S. M. et al., 2004). IL-1 and TNFα stimulate the secretion of IL-6, which in turn exerts negative feedback regulation on the production of IL-1 and TNFα (O’Brien S. M. et al., 2004). IL-6, IL-1β, and TNFα stimulate the secretion of CRF and results in hyperactivity of the HPA axis (Dentino et al., 1999; Kariagina et al., 2004). A CRF1 antagonist (SSR125543) can block the effects of inflammatory cytokines on stress-related behaviors (Knapp et al., 2011). Moreover, CRF can induce the release of TNF-α in glial cells (Wang et al., 2003). Another study demonstrated that intraperitoneal injection of CRF increased the expression of TNF-α and IL-6 (Chen H. et al., 2018). These results imply that during depression, proinflammatory cytokines stimulate the secretion of CRF, and CRF activation may in turn facilitate secretion of proinflammatory cytokines. Anti-inflammatory cytokines play different roles in CRF-driven regulation of depression. IL-10, an anti-inflammatory cytokine produced in lymphocytes and CNS structures such as pituitary and hypothalamus, plays a key role in limiting immune responses and further inhibiting the production of cytokines (Smith et al., 1999; Kiecolt-Glaser and Glaser, 2002). IL-10 attenuates the proinflammatory state produced by LPS (Hennessy et al., 2011). In clinical studies, patients with depression treated with four antidepressants (venlafaxine, L-5-hydroxytryptophan, fluoxetine, and imipramine) showed increase in the production of IL-10 (Kubera et al., 2000, 2001). Under conditions of stress, IL-10 production by lymphocytes or hypothalamus is increased along with the levels of ACTH and CRF (Smith et al., 1999). IL-10 has been suggested to prevent the passive behavior caused by CRF injection (Hennessy et al., 2011). As IL-10 can stimulate the secretion of ACTH, this preventive effect may be partly due to the ACTH mediated short feedback loop inhibition of CRF (Smith et al., 1999). Another clinical study showed that CRF treatment suppresses IL-10 production in both Alzheimer’s disease (AD) patients and healthy controls, and this process was regulated by T cells (Oh et al., 2012). Both proinflammatory and anti-inflammatory cytokines can enhance the production of CRF. However, the effects of CRF on proinflammatory and anti-inflammatory cytokines are opposite. CRF stimulates the secretion of proinflammatory cytokines while it suppresses the secretion of anti-inflammatory cytokines. Taken together, interactions between CRF and cytokines play a crucial role in the pathology of depression and targeting the network of cytokines and CRF may be an effective therapeutic strategy for this mood disorder.

Peripheral immune cells such as T cells play an important role in the stress-induced immune response (Haczku and Panettieri, 2010). The immunomodulatory effect of CRF is not restricted to the nervous system as CRF also exerts peripheral regulatory effects on skin, the gastrointestinal tract and the cardiovascular system (Slominski et al., 2013). CRF receptors are expressed by a variety of immune cells, such as mast cells, dendritic cells, B cells, and T cells (Chatoo et al., 2018; Harle et al., 2018). Chronic exposure to CRF and glucocorticoids results in immune dysregulation such as a reduction in T-cell proliferation (Oh et al., 2012; Jin et al., 2016). One primary function of T cells in the immune system is to produce cytokines. CRF suppresses the anti-inflammatory cytokine IL-10 in regulatory T (Treg) cells, a kind of T cells that contribute to stress-related exacerbation in AD (Oh et al., 2012). A recent study demonstrated that CRF can disturb the immunosuppressive effect of Treg cells on CD4^+^ T cells via suppressing a protein named dedicator of cytokinesis 8 (DOCK8), and this effect may contribute to stress-induced aggravation of AD (Jin et al., 2016). Interestingly, lymphocytes like T cells, and B cells also have the ability to secrete CRF (Kravchenco and Furalev, 1994). The interactions between T cells and CRF in depression are yet to be explored.

Accumulating evidence suggests that glial cells, a major cellular component of the neuroimmune system, are also involved in the pathology of depression. Oligodendrocytes, astrocytes, and microglia are some of the most common types of glial cells in the CNS (Miller and O’Callaghan, 2005). Loss of glial cells in amygdala and subgenual prefrontal cortex has been reported in depressed subjects (Ongur et al., 1998; Hamidi et al., 2004). A decrease in expression of GFAP, a marker of astrocytes, was observed in depression patients (Miguel-Hidalgo et al., 2000). In addition, glial ablation in the pre-frontal cortex induced depressive-like behaviors in rats (Banasr and Duman, 2008). These findings suggest a crucial role of glial cells in depression, and glial cell dysfunction may contribute to progression of this disorder. Microglia belongs to macrophage populations, and plays a key role in CNS homeostasis (Perry and Teeling, 2013). Microglia are in a resting state under basal conditions. They can undergo morphological changes and modulate into phagocytic cells once activated (Vilhardt, 2005). Activated microglia and astrocytes produce pro-inflammatory cytokines such as TNFα, IL-1, and IL-6, resulting in neuroinflammation (Lee et al., 2000; Zhu et al., 2010). Intracerebroventricular administration of LPS induced an up-regulation of proinflammatory cytokines along with an increase in reactive glial markers, and resulted in depressive-like behaviors (Huang et al., 2008). In the CNS, inflammasomes regulate neuroinflammation by mediating the maturation and secretion of pro-inflammatory cytokines (Singhal et al., 2014). Activation of inflammasomes has been found in depression patients (Alcocer-Gomez and Cordero, 2014). In depressed rats, proinflammatory cytokine-related inflammation is mediated by nucleotide binding oligomerization domain-like receptor family pyrin domain-containing 3 (NLRP3) inflammasome (Pan et al., 2014). Chronic stress failed to induce depressive behaviors in the absence of NLRP3 inflammasome (Alcocer-Gomez et al., 2016). Activation of NLRP3 inflammasome in glial cells could also induce depressive-like behaviors in rats (Yue et al., 2017). Furthermore, glial cells mediate the neuroinflammatory process and are involved in the pathogenesis of depression (You et al., 2017). Both CRF1 and CRF2 receptors are expressed in both microglia and astrocytes (Stevens et al., 2003). The activation of microglia and astrocytes in neuroinflammation is mediated by CRF, and this process may be a underlying mechanism of several neurological diseases, including depression (Kritas et al., 2014). Abnormalities in oligodendrocytes have been described in several mood disorders, such as schizophrenia, bipolar disorder, and depression (Aston et al., 2005). A reduction in total glial cells and oligodendrocytes has been found in amygdala of the brains of depressed subjects while no significant difference in astrocytes or microglia density was observed (Hamidi et al., 2004). There is no direct evidence of the presence of CRF receptors in oligodendrocytes, but CRF elevates cyclic adenosine monophosphate (cAMP) level in these cells (Wiemelt et al., 2001). Thus, CRF receptors may also be expressed in oligodendrocytes as CRF1 is the primary mediator of increase in cAMP in response to CRF stimulation (Stevens et al., 2003). Further studies are needed to elucidate the relationship between oligodendrocytes and CRF.

**FIGURE 1:**
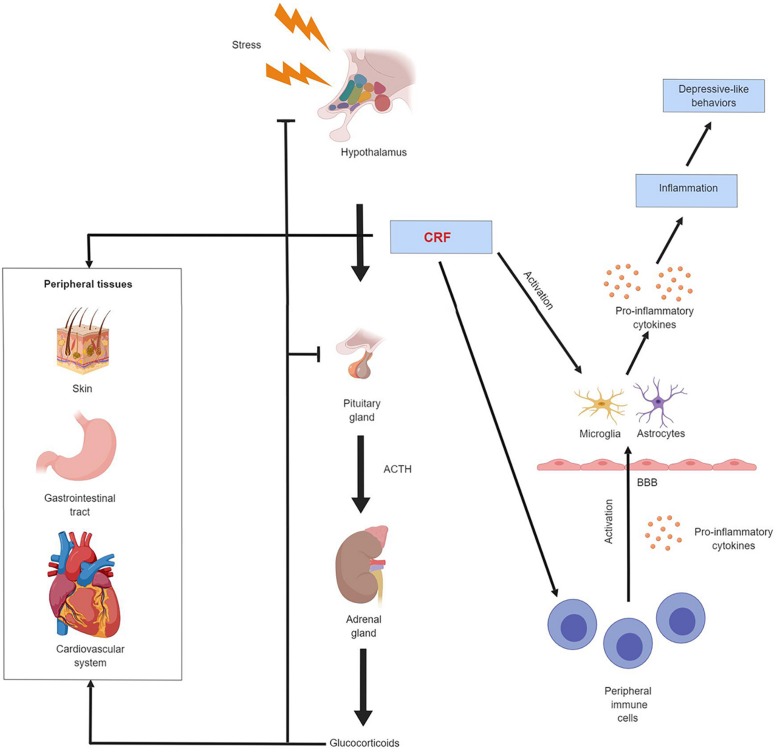
Schematic illustration of CRF regulation of the endocrine and immune system in depression. CRF mediates the activity of the HPA axis and the neuroimmune system. It also exerts regulatory effect on other peripheral tissues such as skin, gastrointestinal tract and cardiovascular system. Chronic exposure to stress results in CRF hypersecretion and HPA axis hyperactivity. Elevated CRF level stimulates the production of pro-inflammatory cytokines by peripheral immune cells, these peripheral cytokines can cross the blood-brain barrier and activate astrocytes and microglia in the CNS. CRF can also directly activate astrocytes and microglia. The activated astrocytes and microglia secrete more pro-inflammatory cytokines. These astrocytes- and microglia-derived cytokines have a broad effect on the CNS, drive neuroinflammation and produce depression-like behavioral alterations.

Cumulatively, CRF regulates the immune responses in the CNS by mediating cytokine production and activation of peripheral immune cells and glial cells (Figure 1). The CRF-mediated immune responses play a crucial role in the pathogenesis of a series of neurological diseases, including depression. However, a recent study reported that chronic high-dose captopril (CHC) administration can induce a specific form of depressive-like behavior. This effect is caused by Treg reduction and microglial activation with unaltered CRF levels and HPA axis activity (Park et al., 2017). This finding suggests that the activation of immune cells as a response to depression can also be independent of CRF and the HPA axis regulation.

## Potential Application of Chinese Herbal Medicines in Treating Depression

The links between immune responses and depression have inspired the application of anti-inflammation therapies in the treatment of depression. In depressed patients who also suffer from coronary artery disease, statin treatment can downregulate IL-1β expression and function as an anti-inflammation therapy of depression (Ma et al., 2016). Another study demonstrated that chronic treatment with the non-steroidal anti-inflammatory drug (NSAID), celecoxib, reversed the depressive-like behavior in stressed rats by inhibiting cyclooxygenase (COX)-2 expression (Guo et al., 2009). Ginseng total saponins (GTS) are effective in attenuating lipopolysaccharide- (LPS) induced depression-like behavior because of its peripheral anti-inflammatory activity (Kang et al., 2011). Ethyleicosapentaenoate (EPA) has been used to treat depression, and such an activity likely originates from suppression of inflammation and upregulation of nerve growth factor (NGF) (Song et al., 2009). Besides the use of drugs, other approaches that suppress inflammation may also be a potential treatment strategy for depression. A recent clinical study suggests that transcutaneous auricular vagus nerve stimulation (taVNS) can alleviate multiple symptoms of depression and one of the possible underlying mechanisms is that taVNS may inhibit inflammatory responses and relieve stress (Kong et al., 2018).

It is worth noting that many CHM have been long used for anti-inflammatory properties. The biologically active components of CHM has been reported to inhibit proinflammatory pathways (Pan et al., 2011). Anti-depression effects have been found in a vast number of CHM such as Tianshu capsule, Danggui-Shaoyao-San, and Kai-Xin-San (Xu et al., 2011; Zhu et al., 2016; Sun et al., 2018). Thus, these CHM hold potential as anti-depression medications. In rats, tribulus terrestris saponins (TTS) treatment significantly reduced chronic mild stress (CMS) induced increase of serum CRF (and CORT) and depressive-like symptoms, which indicates that antidepressant effects of TTS may be attributed to down-regulation of HPA axis hyperactivity by CRF regulation (Wang et al., 2013). Salidroside (SA) showed antidepressant activities in olfactory bulbectomized rats by reversing the elevated CRH expression in hypothalamus and serum CORT level, and the normalization of HPA axis hyperactivity by SA may be due to its anti-inflammatory properties (Yang et al., 2014). Oral administration of saikosaponin A, one of the main constituents of Chai hu, restored the elevated pro-inflammatory cytokines levels and CRF level in depressed rats (Chen X. Q. et al., 2018). However, direct intracerebroventricular injection of saikosaponin A failed to affect CRF levels, while saikosaponin D, another major component of Chai hu, increased CRF mRNA level in the hypothalamus in the same study (Dobashi et al., 1995). Therefore, instead of directly affecting CRF levels, saikosaponin A may regulate CRF levels by suppressing neuroinflammation (Chen X. Q. et al., 2018). These findings suggest that antidepressants, including CHM can restore HPA axis hyperactivity by decreasing CRF levels, and such effect may be due to direct regulation of CRF levels, or indirect regulation of neuroimmune mechanisms.

Although many CHM have shown promising antidepressant-like effects, their exact mechanisms of action remain unclear. Future studies are needed to find out their direct targets in depression treatment. Depression is a multifactorial disease. Most CHM act through multiple mechanisms simultaneously. Therefore, they have advantages over other single-target drugs in depression treatment. In addition, the compatibility of CHM may have better therapeutic effects than using a single drug in treating complex diseases, such as depression. Developing novel plant-based medicines against depression is an important imperative to strengthen the public health and enrich our knowledge about the potential use and value of CHM.

## CRF1 Antagonists and Other Antidepressants

Corticotropin releasing factor exerts its effect on various tissues via acting on CRF receptors. As CRF/CRF1 signaling involved in the pathogenesis of depression, blocking CRF1 receptor may be an effective therapeutic approach. Several CRF1 receptor specific antagonists with potent antidepressant-like effects have been developed (Zoumakis et al., 2006). For example, a selective CRF1 receptor antagonist E2508 shortened immobility time in the rat forced swim test (Taguchi et al., 2016). Besides treating depression, CRF1 receptor antagonists may have many other applications because of the multifaceted actions of CRF/CRF1 system. For example, potential clinical applications of CRF1 receptor antagonists include the treatment of anxiety, allergy, autoimmune inflammatory disorders, epilepsy and so on (Grammatopoulos and Chrousos, 2002). In aged rats, two CRF1 receptor antagonists, R121919 and antalarmin, prevented chronic stress-induced anxiety-related behavioral and memory deficits (Dong et al., 2018). Although CRF1 receptor antagonists show promising effects in rodents, their clinical efficacy is mixed. GSK561679, BMS-562086, GSK561679, and GW-876008 yield negative results in clinical trials in patients with depression and anxiety disorders (Griebel and Holsboer, 2012; Dunlop et al., 2017). In contrast, two clinical trials with pexacerfont and verucerfont showed positive effects in treating withdrawal symptoms and stress-induced alcohol craving (Schwandt et al., 2016; Morabbi et al., 2018). One possible reason of these failures might be the heterogenous response to CRF1 receptor antagonists treatment (Licinio et al., 2004). These individual differences may be caused by genetic variability of *CRHR1*, the gene encoding for CRF1 receptor, or different activity in CRF-CRF1 systems (Spierling and Zorrilla, 2017; Davis et al., 2018). Further studies can focus on developing personalized treatment plans for depression. Evaluating genetic or non-genetic markers may aid in developing specific CRF1 antagonists for specific patient subgroups. Besides CRF1 receptor antagonists, activation of CRF2 with two selective agonists, urocortin 2 (UCN 2) and urocortin 3 (UCN 3), reversed depression- and anxiety-like behaviors (Bagosi et al., 2016). The development of selective antagonists of CRF1 receptor or agonists of CRF2 receptor may aid in developing novel treatments to a wide array of stress-related diseases, including depression.

Several other antidepressants have been used for the treatment of depression, such as triple uptake inhibitors, monoamine oxidase inhibitors and selective monoamine reuptake inhibitors (de Oliveira et al., 2018). Overall, the efficiency and the therapeutic window of anti-depressants are limited. Only about 50% of all patients receiving anti-depressants have complete remission (Nestler et al., 2002). Moreover, the mechanism of action of anti-depressants is usually much more complex than expected. As a result, anti-depressant medications generally cause a variety of side effects. Therefore, it is extremely important to develop novel anti-depressants having high efficiency and less side effects.

## Conclusion

Depression is a very complex neurological disorder. The normal functioning of the brain is carried out by intricate interactions between CNS and peripheral systems such as gastrointestinal tract, cardiovascular system, and immune system. Dysregulation of any key mediators in these systems may break the homeostasis and subsequently result in neurological diseases. CRF affects the various biological processes in human body, and an increasing volume of data suggests a crucial role for CRF in the immune regulation of depression. CRF is a key regulator of the HPA axis, which is a common pathway of stress response involved in the pathogenesis of a variety of neurological diseases and it can also regulate the neuroimmune system by mediating cytokine production and neuroinflammation. CRF receptors are expressed in peripheral immune cells, glial cells and neurons. Dysregulation of CRF caused by external and internal factors can result in neuronal and endocrinal consequences and drives depressive behaviors. It is notable that bidirectional regulation is a common feature of the interactions between CRF, immune cells and cytokines. Further studies are required to establish a deeper understanding of the complex network of CRF-mediated immune crosstalk in depression.

In conclusion, this review provides a basis for the crucial role of CRF in the neuroimmune regulation of depression. Studying the interaction of CRF and immune responses can help enhance our understanding of the pathogenesis of depression. Furthermore, targeting this network may facilitate new therapeutic approaches to counteract depression, and other stress-related diseases.

## Author Contributions

YJ and TP drafted and wrote the manuscript. YJ, TP, UG, MS, PL, WQ, ZC, YZ, and WZ did the critical revision of the manuscript.

## Conflict of Interest Statement

The authors declare that the research was conducted in the absence of any commercial or financial relationships that could be construed as a potential conflict of interest.

## References

[B1] Alcocer-Gomez E. Cordero M. D. (2014). NLRP3 inflammasome: a new target in major depressive disorder. *CNS Neurosci. Ther.* 20 294–295. 10.1111/cns.12230 24479787 PMC6493047

[B2] Alcocer-Gomez E. Ulecia-Moron C. Marin-Aguilar F. Rybkina T. Casas-Barquero N. Ruiz-Cabello J. (2016). Stress-induced depressive behaviors require a functional NLRP3 inflammasome. *Mol. Neurobiol.* 53 4874–4882. 10.1007/s12035-015-9408-7 26362308

[B3] American Psychiatric Association (2013). *Diagnostic and Statistical Manual of Mental Disorders* (5th ed). Arlington, VA: American Psychiatric Publishing.

[B4] Ancelin M. L. Scali J. Norton J. Ritchie K. Dupuy A. M. Chaudieu I. Ryan J. (2017). Heterogeneity in HPA axis dysregulation and serotonergic vulnerability to depression. *Psychoneuroendocrinology* 77 90–94. 10.1016/j.psyneuen.2016.11.016 28024274

[B5] Arborelius L. Owens M. J. Plotsky P. M. Nemeroff C. B. (1999). The role of corticotropin-releasing factor in depression and anxiety disorders. *J. Endocrinol.* 160 1–12. 10.1677/joe.0.1600001 9854171

[B6] Aston C. Jiang L. Sokolov B. P. (2005). Transcriptional profiling reveals evidence for signaling and oligodendroglial abnormalities in the temporal cortex from patients with major depressive disorder. *Mol. Psychiatry* 10 309–322. 10.1038/sj.mp.4001565 15303102

[B7] Bagosi Z. Palotai M. Simon B. Bokor P. Buzas A. Balango B. (2016). Selective CRF2 receptor agonists ameliorate the anxiety- and depression-like state developed during chronic nicotine treatment and consequent acute withdrawal in mice. *Brain Res.* 1652 21–29. 10.1016/j.brainres.2016.09.044 27693397

[B8] Bale T. L. Vale W. W. (2003). Increased depression-like behaviors in corticotropin-releasing factor receptor-2-deficient mice: sexually dichotomous responses. *J. Neurosci.* 23 5295–5301. 10.1523/jneurosci.23-12-05295.2003 12832554 PMC6741155

[B9] Banasr M. Duman R. S. (2008). Glial loss in the prefrontal cortex is sufficient to induce depressive-like behaviors. *Biol. Psychiatry* 64 863–870. 10.1016/j.biopsych.2008.06.008 18639237 PMC2709733

[B10] Bangasser D. A. Wiersielis K. R. Khantsis S. (2016). Sex differences in the locus coeruleus-norepinephrine system and its regulation by stress. *Brain Res*. 1641(Pt B), 177–188. 10.1016/j.brainres.2015.11.021 26607253 PMC4875880

[B11] Bao A. M. Ruhe H. G. Gao S. F. Swaab D. F. (2012). Neurotransmitters and neuropeptides in depression. *Handb. Clin. Neurol.* 106 107–136. 10.1016/B978-0-444-52002-9.00008-5 22608619

[B12] Bao A. M. Swaab D. F. (2010). Corticotropin-releasing hormone and arginine vasopressin in depression focus on the human postmortem hypothalamus. *Vitam. Horm.* 82 339–365. 10.1016/S0083-6729(10)82018-7 20472147

[B13] Beardsley P. M. Hauser K. F. (2014). Glial modulators as potential treatments of psychostimulant abuse. *Adv. Pharmacol*. 69 1–69. 10.1016/B978-0-12-420118-7.00001-9 24484974 PMC4103010

[B14] Beery A. K. Bicks L. Mooney S. J. Goodwin N. L. Holmes M. M. (2016). Sex, social status, and CRF receptor densities in naked mole-rats. *J. Comp. Neurol.* 524 228–243. 10.1002/cne.23834 26100759

[B15] Belmaker R. H. Agam G. (2008). Major depressive disorder. *N. Engl. J. Med.* 358 55–68. 10.1056/NEJMra073096 18172175

[B16] Belvederi Murri M. Pariante C. Mondelli V. Masotti M. Atti A. R. (2014). HPA axis and aging in depression: systematic review and meta-analysis. *Psychoneuroendocrinology* 41 46–62. 10.1016/j.psyneuen.2013.12.004. 24495607

[B17] Berardelli R. Karamouzis I. D’Angelo V. Zichi C. Fussotto B. Giordano R. (2013). Role of mineralocorticoid receptors on the hypothalamus-pituitary-adrenal axis in humans. *Endocrine* 43 51–58. 10.1007/s12020-012-9750-8 22836869

[B18] Bradley S. M. Rumsfeld J. S. (2015). Depression and cardiovascular disease. *Trends Cardiovasc. Med.* 25 614–22. 10.1016/j.tcm.2015.02.002 25850976

[B19] Bremmer M. A. Deeg D. J. Beekman A. T. Penninx B. W. Lips P. Hoogendijk W. J. (2007). Major depression in late life is associated with both hypo- and hypercortisolemia. *Biol. Psychiatry* 62 479–486. 10.1016/j.biopsych.2006.11.033 17481591

[B20] Chatoo M. Li Y. Ma Z. Coote J. Du J. (2018). Involvement of corticotropin-releasing factor and receptors in immune cells in irritable bowel syndrome. *Front. Endocrinol.* 9:21 10.3389/fendo.2018.00021 PMC5816029 29483895

[B21] Chen H. Shi H. T. Liu Y. P. Ren X. Y. He S. X. Chang X. M. Yin Y. (2018). Activation of corticotropin-releasing factor receptor 1 aggravates dextran sodium sulphate-induced colitis in mice by promoting M1 macrophage polarization. *Mol. Med. Rep.* 17 234–242. 10.3892/mmr.2017.7909 29115460 PMC5780132

[B22] Chen L. Li S. Cai J. Wei T. J. Liu L. Y. Zhao H. Y. (2018). Activation of CRF/CRFR1 signaling in the basolateral nucleus of the amygdala contributes to chronic forced swim-induced depressive-like behaviors in rats. *Behav. Brain Res.* 338 134–142. 10.1016/j.bbr.2017.10.027 29080675

[B23] Chen X. Q. Chen S. J. Liang W. N. Wang M. Li C. F. Wang S. S. (2018). Saikosaponin A attenuates perimenopausal depression-like symptoms by chronic unpredictable mild stress. *Neurosci. Lett.* 662 283–289. 10.1016/j.neulet.2017.09.046 28958685

[B24] Christoffel D. J. Golden S. A. Russo S. J. (2011). Structural and synaptic plasticity in stress-related disorders. *Rev. Neurosci.* 22 535–549. 10.1515/RNS.2011.044 21967517 PMC3212803

[B25] Coste S. C. Kesterson R. A. Heldwein K. A. Stevens S. L. Heard A. D. Hollis J. H. (2000). Abnormal adaptations to stress and impaired cardiovascular function in mice lacking corticotropin-releasing hormone receptor-2. *Nat. Genet.* 24 403–409. 10.1038/74255 10742107

[B26] Davidson R. J. McEwen B. S. (2012). Social influences on neuroplasticity: stress and interventions to promote well-being. *Nat. Neurosci.* 15 689–695. 10.1038/nn.3093 22534579 PMC3491815

[B27] Davis E. G. Keller J. Hallmayer J. Pankow H. R. Murphy G. M. Jr. Gotlib I. H. (2018). Corticotropin-releasing factor 1 receptor haplotype and cognitive features of major depression. *Transl. Psychiatry* 8:5. 10.1038/s41398-017-0051-0 29317606 PMC5802461

[B28] de Kloet E. R. Joels M. Holsboer F. (2005). Stress and the brain: from adaptation to disease. *Nat. Rev. Neurosci.* 6 463–475. 10.1038/nrn1683 15891777

[B29] de Oliveira M. R. Chenet A. L. Duarte A. R. Scaini G. Quevedo J. (2018). Molecular mechanisms underlying the anti-depressant effects of resveratrol: a review. *Mol. Neurobiol.* 55 4543–4559. 10.1007/s12035-017-0680-6 28695536

[B30] Dentino A. N. Pieper C. F. Rao M. K. Currie M. S. Harris T. Blazer D. G. (1999). Association of interleukin-6 and other biologic variables with depression in older people living in the community. *J. Am. Geriatr. Soc.* 47 6–11. 10.1111/j.1532-5415.1999.tb01894.x 9920223

[B31] Dobashi I. Tozawa F. Horiba N. Sakai Y. Sakai K. Suda T. (1995). Central administration of saikosaponin-d increases corticotropin-releasing factor mRNA levels in the rat hypothalamus. *Neurosci. Lett.* 197 235–238. 10.1016/0304-3940(95)11933-n 8552307

[B32] Dong H. Keegan J. M. Hong E. Gallardo C. Montalvo-Ortiz J. Wang B. (2018). Corticotrophin releasing factor receptor 1 antagonists prevent chronic stress-induced behavioral changes and synapse loss in aged rats. *Psychoneuroendocrinology* 90 92–101. 10.1016/j.psyneuen.2018.02.013 29477954 PMC5864558

[B33] Dowlati Y. Herrmann N. Swardfager W. Liu H. Sham L. Reim E. K. Lanctot K. L. (2010). A meta-analysis of cytokines in major depression. *Biol. Psychiatry* 67 446–457. 10.1016/j.biopsych.2009.09.033 20015486

[B34] Duman R. S. (2014). Neurobiology of stress, depression, and rapid acting antidepressants: remodeling synaptic connections. *Depress. Anxiety* 31 291–296. 10.1002/da.22227 24616149 PMC4432471

[B35] Duman R. S. Aghajanian G. K. Sanacora G. Krystal J. H. (2016). Synaptic plasticity and depression new insights from stress and rapid-acting antidepressants. *Nat. Med.* 22 238–49. 10.1038/nm.4050 26937618 PMC5405628

[B36] Dunlop B. W. Binder E. B. Iosifescu D. Mathew S. J. Neylan T. C. Pape J. C. (2017). Corticotropin-releasing factor receptor 1 antagonism is ineffective for women with posttraumatic stress disorder. *Biol. Psychiatry* 82 866–874. 10.1016/j.biopsych.2017.06.024 28793974 PMC5683912

[B37] Erickson M. A. Dohi K. Banks W. A. (2012). Neuroinflammation: a common pathway in CNS diseases as mediated at the blood-brain barrier. *Neuroimmunomodulation* 19 121–130. 10.1159/000330247 22248728 PMC3707010

[B38] Evans D. L. Charney D. S. Lewis L. Golden R. N. Gorman J. M. Krishnan K. R. (2005). Mood disorders in the medically ill: scientific review and recommendations. *Biol. Psychiatry* 58 175–189. 10.1016/j.biopsych.2005.05.001 16084838

[B39] Ferrari E. Casarotti D. Muzzoni B. Albertelli N. Cravello L. Fioravanti M. (2001). Age-related changes of the adrenal secretory pattern: possible role in pathological brain aging. *Brain Res. Brain Res. Rev.* 37 294–300. 10.1016/s0165-0173(01)00133-3 11744094

[B40] GBD (2015). Disease and injury incidence and prevalence collaborators (2016). Global, regional, and national incidence, prevalence, and years lived with disability for 310 diseases and injuries, 1990-2015: a systematic analysis for the global burden of disease study 2015. *Lancet* 388 1545–1602. 10.1016/S0140-6736(16)31678-6 PMC5055577 27733282

[B41] Gimsa U. Mitchison N. A. Brunner-Weinzierl M. C. (2013). Immune privilege as an intrinsic CNS property: astrocytes protect the CNS against T-Cell-mediated neuroinflammation. *Mediators Inflamm.* 2013:320519. 10.1155/2013/320519 24023412 PMC3760105

[B42] Gotthardt U. Schweiger U. Fahrenberg J. Lauer C. J. Holsboer F. (1995). Cortisol, ACTH, and cardiovascular response to a cognitive challenge paradigm in aging and depression. *Am. J. Physiol.* 268(4 Pt 2), R865–R873. 10.1152/ajpregu.1995.268.4.R865 7733395

[B43] Grammatopoulos D. K. Chrousos G. P. (2002). Functional characteristics of CRH receptors and potential clinical applications of CRH-receptor antagonists. *Trends Endocrinol. Metab.* 13 436–444. 10.1016/s1043-2760(02)00670-7 12431840

[B44] Greetfeld M. Schmidt M. V. Ganea K. Sterlemann V. Liebl C. Muller M. B. (2009). A single episode of restraint stress regulates central corticotrophin- releasing hormone receptor expression and binding in specific areas of the mouse brain. *J. Neuroendocrinol.* 21 473–480. 10.1111/j.1365-2826.2009.01865.x 19302188

[B45] Griebel G. Holsboer F. (2012). Neuropeptide receptor ligands as drugs for psychiatric diseases: the end of the beginning? *Nat. Rev. Drug Discov.* 11 462–478. 10.1038/nrd3702 22596253

[B46] Guo J. Y. Li C. Y. Ruan Y. P. Sun M. Qi X. L. Zhao B. S. (2009). Chronic treatment with celecoxib reverses chronic unpredictable stress-induced depressive-like behavior via reducing cyclooxygenase-2 expression in rat brain. *Eur. J. Pharmacol.* 612 54–60. 10.1016/j.ejphar.2009.03.076 19356723

[B47] Haczku A. Panettieri R. A. Jr. (2010). Social stress and asthma: the role of corticosteroid insensitivity. *J. Allergy Clin. Immunol.* 125 550–558. 10.1016/j.jaci.2009.11.005 20153032 PMC2839059

[B48] Hamidi M. Drevets W. C. Price J. L. (2004). Glial reduction in amygdala in major depressive disorder is due to oligodendrocytes. *Biol. Psychiatry* 55 563–569. 10.1016/j.biopsych.2003.11.006 15013824

[B49] Harle G. Kaminski S. Dubayle D. Frippiat J. P. Ropars A. (2018). Murine splenic B cells express corticotropin-releasing hormone receptor 2 that affect their viability during a stress response. *Sci. Rep.* 8:143. 10.1038/s41598-017-18401-y 29317694 PMC5760685

[B50] Hashimoto D. Miller J. Merad M. (2011). Dendritic cell and macrophage heterogeneity in vivo. *Immunity* 35 323–335. 10.1016/j.immuni.2011.09.007 21943488 PMC4520532

[B51] Hennessy M. B. Fitch C. Jacobs S. Deak T. Schiml P. A. (2011). Behavioral effects of peripheral corticotropin-releasing factor during maternal separation may be mediated by proinflammatory activity. *Psychoneuroendocrinology* 36 996–1004. 10.1016/j.psyneuen.2010.12.011 21255937 PMC3568995

[B52] Herbert T. B. Cohen S. (1993). Depression and immunity: a meta-analytic review. *Psychol. Bull.* 113 472–486. 10.1037//0033-2909.113.3.472 8316610

[B53] Hodes G. E. Kana V. Menard C. Merad M. Russo S. J. (2015). Neuroimmune mechanisms of depression. *Nat. Neurosci.* 18 1386–1393. 10.1038/nn.4113 26404713 PMC4843114

[B54] Holsboer F. (2001). Stress, hypercortisolism and corticosteroid receptors in depression: implications for therapy. *J. Affect. Disord.* 62 77–91. 10.1016/s0165-0327(00)00352-9 11172875

[B55] Holsboer F. Spengler D. Heuser I. (1992). The role of corticotropin-releasing hormone in the pathogenesis of Cushing’s disease, anorexia nervosa, alcoholism, affective disorders and dementia. *Prog. Brain Res.* 93 385–417. 10.1016/s0079-6123(08)64586-0 1336204

[B56] Hopkins S. J. Rothwell N. J. (1995). Cytokines and the nervous system. I: expression and recognition. *Trends Neurosci.* 18 83–88. 10.1016/0166-2236(95)93881-w 7537419

[B57] Howren M. B. Lamkin D. M. Suls J. (2009). Associations of depression with C-reactive protein, IL-1, and IL-6: a meta-analysis. *Psychosom. Med.* 71 171–186. 10.1097/PSY.0b013e3181907c1b 19188531

[B58] Huang Y. Henry C. J. Dantzer R. Johnson R. W. Godbout J. P. (2008). Exaggerated sickness behavior and brain proinflammatory cytokine expression in aged mice in response to intracerebroventricular lipopolysaccharide. *Neurobiol. Aging* 29 1744–1753. 10.1016/j.neurobiolaging.2007.04.012 17543422 PMC2647751

[B59] Iniguez S. D. Riggs L. M. Nieto S. J. Dayrit G. Zamora N. N. Shawhan K. L. (2014). Social defeat stress induces a depression-like phenotype in adolescent male c57BL/6 mice. *Stress* 17 247–255. 10.3109/10253890.2014.910650 24689732 PMC5534169

[B60] Jin S. Shin J. U. Noh J. Y. Kim H. Kim J. Y. Kim S. H. (2016). DOCK8: regulator of Treg in response to corticotropin-releasing hormone. *Allergy* 71 811–819. 10.1111/all.12845 26799599

[B61] Kang A. Hao H. P. Zheng X. Liang Y. Xie Y. Xie T. (2011). Peripheral anti-inflammatory effects explain the ginsenosides paradox between poor brain distribution and anti-depression efficacy. *J. Neuroinflamm.* 8:100. 10.1186/1742-2094-8-100 21843370 PMC3169467

[B62] Kariagina A. Romanenko D. Ren S. G. Chesnokova V. (2004). Hypothalamic-pituitary cytokine network. *Endocrinology* 145 104–112. 10.1210/en.2003-0669 14512435

[B63] Keck M. E. (2006). Corticotropin-releasing factor, vasopressin and receptor systems in depression and anxiety. *Amino Acids* 31 241–250. 10.1007/s00726-006-0333-y 16733617

[B64] Kessler R. C. McGonagle K. A. Swartz M. Blazer D. G. Nelson C. B. (1993). Sex and depression in the national comorbidity survey. I: lifetime prevalence, chronicity and recurrence. *J. Affect. Disord.* 29 85–96. 10.1016/0165-0327(93)90026-g 8300981

[B65] Ketchesin K. D. Stinnett G. S. Seasholtz A. F. (2017). Corticotropin-releasing hormone-binding protein and stress: from invertebrates to humans. *Stress* 20 449–464. 10.1080/10253890.2017.1322575 28436309 PMC7885796

[B66] Kiecolt-Glaser J. K. Glaser R. (2002). Depression and immune function: central pathways to morbidity and mortality. *J. Psychosom. Res.* 53 873–876. 12377296 10.1016/s0022-3999(02)00309-4

[B67] Kinlein S. A. Wilson C. D. Karatsoreos I. N. (2015). Dysregulated hypothalamic-pituitary-adrenal axis function contributes to altered endocrine and neurobehavioral responses to acute stress. *Front. Psychiatry* 6:31. 10.3389/fpsyt.2015.00031 25821436 PMC4358064

[B68] Knapp D. J. Whitman B. A. Wills T. A. Angel R. A. Overstreet D. H. Criswell H. E. (2011). Cytokine involvement in stress may depend on corticotrophin releasing factor to sensitize ethanol withdrawal anxiety. *Brain Behav. Immun.* 25 S146–S154. 10.1016/j.bbi.2011.02.018 21377524 PMC3138123

[B69] Kong J. Fang J. Park J. Li S. Rong P. (2018). Treating depression with transcutaneous auricular vagus nerve stimulation: state of the art and future perspectives. *Front. Psychiatry* 9:20. 10.3389/fpsyt.2018.00020 29459836 PMC5807379

[B70] Koutmani Y. Politis P. K. Elkouris M. Agrogiannis G. Kemerli M. Patsouris E. (2013). Corticotropin-releasing hormone exerts direct effects on neuronal progenitor cells: implications for neuroprotection. *Mol. Psychiatry* 18 300–307. 10.1038/mp.2012.198 23380766 PMC3578949

[B71] Kravchenco I. V. Furalev V. A. (1994). Secretion of immunoreactive corticotropin releasing factor and adrenocorticotropic hormone by T- and B-lymphocytes in response to cellular stress factors. *Biochem. Biophys. Res. Commun.* 204 828–834. 10.1006/bbrc.1994.2534 7980549

[B72] Kritas S. K. Saggini A. Cerulli G. Caraffa A. Antinolfi P. Pantalone A. (2014). Corticotropin-releasing hormone, microglia and mental disorders. *Int. J. Immunopathol. Pharmacol.* 27 163–167. 10.1177/039463201402700203 25004828

[B73] Kubera M. Kenis G. Bosmans E. Scharpe S. Maes M. (2000). Effects of serotonin and serotonergic agonists and antagonists on the production of interferon-gamma and interleukin-10. *Neuropsychopharmacology* 23 89–98. 10.1016/S0893-133X(99)00150-5 10869889

[B74] Kubera M. Lin A. H. Kenis G. Bosmans E. van Bockstaele D. (2001). Anti-inflammatory effects of antidepressants through suppression of the interferon-gamma/interleukin-10 production ratio. *J. Clin. Psychopharmacol.* 21 199–206. 10.1097/00004714-200104000-00012 11270917

[B75] Lee S. J. Drabik K. Van Wagoner N. J. Lee S. Choi C. Dong Y. (2000). ICAM-1-induced expression of proinflammatory cytokines in astrocytes: involvement of extracellular signal-regulated kinase and p38 mitogen-activated protein kinase pathways. *J. Immunol.* 165 4658–4666. 10.4049/jimmunol.165.8.4658 11035109

[B76] Li M. D’Arcy C. Meng X. (2016). Maltreatment in childhood substantially increases the risk of adult depression and anxiety in prospective cohort studies: systematic review, meta-analysis, and proportional attributable fractions. *Psychol. Med.* 46 717–730. 10.1017/S0033291715002743 26708271

[B77] Licinio J. O’Kirwan F. Irizarry K. Merriman B. Thakur S. Jepson R. (2004). Association of a corticotropin-releasing hormone receptor 1 haplotype and antidepressant treatment response in Mexican-Americans. *Mol. Psychiatry* 9 1075–1082. 10.1038/sj.mp.4001587 15365580

[B78] Lightman S. L. (2008). The neuroendocrinology of stress: a never ending story. *J. Neuroendocrinol.* 20 880–884. 10.1111/j.1365-2826.2008.01711.x 18601712

[B79] Lu A. Steiner M. A. Whittle N. Vogl A. M. Walser S. M. Ableitner M. (2008). Conditional mouse mutants highlight mechanisms of corticotropin-releasing hormone effects on stress-coping behavior. *Mol. Psychiatry* 13 1028–1042. 10.1038/mp.2008.51 18475271

[B80] Ma W. Shen D. Liu J. Pan J. Yu L. Shi W. (2016). Statin function as an anti-inflammation therapy for depression in patients with coronary artery disease by downregulating interleukin-1beta. *J. Cardiovasc. Pharmacol.* 67 129–135. 10.1097/FJC.0000000000000323 26398164

[B81] Maes M. Van der Planken M. Stevens W. J. Peeters D. DeClerck L. S. Bridts C. H. (1992). Leukocytosis, monocytosis and neutrophilia: hallmarks of severe depression. *J. Psychiatr. Res.* 26 125–134. 10.1016/0022-3956(92)90004-8 1613679

[B82] McEwen B. S. (2007). Physiology and neurobiology of stress and adaptation: central role of the brain. *Physiol. Rev.* 87 873–904. 10.1152/physrev.00041.2006 17615391

[B83] Miguel-Hidalgo J. J. Baucom C. Dilley G. Overholser J. C. Meltzer H. Y. Stockmeier C. A. (2000). Glial fibrillary acidic protein immunoreactivity in the prefrontal cortex distinguishes younger from older adults in major depressive disorder. *Biol. Psychiatry* 48 861–873. 10.1016/S0006-3223(00)00999-9 11063981

[B84] Miller D. B. O’Callaghan J. P. (2005). Depression, cytokines, and glial function. *Metabolism* 54(5 Suppl 1), 33–38. 10.1016/j.metabol.2005.01.011 15877311

[B85] Morabbi M. J. Razaghi E. Moazen-Zadeh E. Safi-Aghdam H. Zarrindast M. R. Vousoghi N. (2018). Pexacerfont as a CRF1 antagonist for the treatment of withdrawal symptoms in men with heroin/methamphetamine dependence: a randomized, double-blind, placebo-controlled clinical trial. *Int. Clin. Psychopharmacol.* 33 111–119. 10.1097/Yic.0000000000000200 29064909

[B86] Morrison M. F. Redei E. TenHave T. Parmelee P. Boyce A. A. Sinha P. S. Katz I. R. (2000). Dehydroepiandrosterone sulfate and psychiatric measures in a frail, elderly residential care population. *Biol. Psychiatry* 47 144–150. 10.1016/S0006-3223(99)00099-2 10664831

[B87] Muller M. B. Landgraf R. Preil J. Sillaber I. Kresse A. E. Keck M. E. (2000). Selective activation of the hypothalamic vasopressinergic system in mice deficient for the corticotropin-releasing hormone receptor 1 is dependent on glucocorticoids. *Endocrinology* 141 4262–4269. 10.1210/endo.141.11.7767 11089561

[B88] Muller M. B. Zimmermann S. Sillaber I. Hagemeyer T. P. Deussing J. M. Timpl P. (2003). Limbic corticotropin-releasing hormone receptor 1 mediates anxiety-related behavior and hormonal adaptation to stress. *Nat. Neurosci.* 6 1100–1107. 10.1038/nn1123 12973355

[B89] Murphy B. E. (1991). Steroids and depression. *J. Steroid Biochem. Mol. Biol.* 38 537–559. 1645586 10.1016/0960-0760(91)90312-s

[B90] Naughton M. Dinan T. G. Scott L. V. (2014). Corticotropin-releasing hormone and the hypothalamic-pituitary-adrenal axis in psychiatric disease. *Handb. Clin. Neurol.* 124 69–91. 10.1016/B978-0-444-59602-4.00005-8 25248580

[B91] Nemeroff C. B. Widerlov E. Bissette G. Walleus H. Karlsson I. Eklund K. (1984). Elevated concentrations of CSF corticotropin-releasing factor-like immunoreactivity in depressed patients. *Science* 226 1342–1344. 10.1126/science.6334362 6334362

[B92] Nestler E. J. Barrot M. DiLeone R. J. Eisch A. J. Gold S. J. Monteggia L. M. (2002). Neurobiology of depression. *Neuron* 34 13–25. 11931738 10.1016/s0896-6273(02)00653-0

[B93] O’Brien J. T. Lloyd A. McKeith I. Gholkar A. Ferrier N. (2004). A longitudinal study of hippocampal volume, cortisol levels, and cognition in older depressed subjects. *Am. J. Psychiatry* 161 2081–2090. 10.1176/appi.ajp.161.11.2081 15514410

[B94] O’Brien S. M. Scott L. V. Dinan T. G. (2004). Cytokines: abnormalities in major depression and implications for pharmacological treatment. *Hum. Psychopharmacol.* 19 397–403. 10.1002/hup.609 15303243

[B95] O’Byrne K. J. Dalgleish A. (2001). Chronic immune activation and inflammation as the cause of malignancy. *Br. J. Cancer* 85 473–483. 10.1054/bjoc.2001.1943 11506482 PMC2364095

[B96] Oh S. H. Park C. O. Wu W. H. Kim J. Y. Jin S. Byamba D. (2012). Corticotropin-releasing hormone downregulates IL-10 production by adaptive forkhead box protein 3-negative regulatory T cells in patients with atopic dermatitis. *J. Allergy Clin. Immunol.* 129 151–159.e1-6. 10.1016/j.jaci.2011.09.008 22000570

[B97] Oldehinkel A. J. van den Berg M. D. Flentge F. Bouhuys A. L. ter Horst G. J. Ormel J. (2001). Urinary free cortisol excretion in elderly persons with minor and major depression. *Psychiatry Res.* 104 39–47. 10.1016/S0165-1781(01)00300-6 11600188

[B98] Ongur D. Drevets W. C. Price J. L. (1998). Glial reduction in the subgenual prefrontal cortex in mood disorders. *Proc. Natl. Acad. Sci. U.S.A.* 95 13290–13295. 10.1073/pnas.95.22.13290 9789081 PMC23786

[B99] Otte C. Gold S. M. Penninx B. W. Pariante C. M. Etkin A. Fava M. (2016). Major depressive disorder. *Nat. Rev. Dis. Primers* 2:16065. 10.1038/nrdp.2016.65 27629598

[B100] Pan M. H. Chiou Y. S. Tsai M. L. Ho C. T. (2011). Anti-inflammatory activity of traditional Chinese medicinal herbs. *J. Tradit. Complement. Med.* 1 8–24. 10.1016/s2225-4110(16)30052-9 24716101 PMC3943005

[B101] Pan Y. Chen X. Y. Zhang Q. Y. Kong L. D. (2014). Microglial NLRP3 inflammasome activation mediates IL-1 beta-related inflammation in prefrontal cortex of depressive rats. *Brain Behav. Immun.* 41 90–100. 10.1016/j.bbi.2014.04.007 24859041

[B102] Pan Y. Zhang W. Y. Xia X. Kong L. D. (2006). Effects of icariin on hypothalamic-pituitary-adrenal axis action and cytokine levels in stressed sprague-dawley rats. *Biol. Pharm. Bull.* 29 2399–2403. 10.1248/Bpb.29.2399 17142971

[B103] Park H. S. Han A. Yeo H. L. Park M. J. You M. J. Choi H. J. (2017). Chronic high dose of captopril induces depressive-like behaviors in mice: possible mechanism of regulatory T cell in depression. *Oncotarget* 8 72528–72543. 10.18632/oncotarget.19879 29069807 PMC5641150

[B104] Perry V. H. Teeling J. (2013). Microglia and macrophages of the central nervous system: the contribution of microglia priming and systemic inflammation to chronic neurodegeneration. *Semin. Immunopathol.* 35 601–612. 10.1007/s00281-013-0382-8 23732506 PMC3742955

[B105] Potter E. Sutton S. Donaldson C. Chen R. Perrin M. Lewis K. (1994). Distribution of corticotropin-releasing factor receptor mRNA expression in the rat brain and pituitary. *Proc. Natl. Acad. Sci. U.S.A.* 91 8777–8781. 10.1073/pnas.91.19.8777 8090722 PMC44689

[B106] Raadsheer F. C. Hoogendijk W. J. Stam F. C. Tilders F. J. Swaab D. F. (1994). Increased numbers of corticotropin-releasing hormone expressing neurons in the hypothalamic paraventricular nucleus of depressed patients. *Neuroendocrinology* 60 436–444. 10.1159/000126778 7824085

[B107] Raadsheer F. C. van Heerikhuize J. J. Lucassen P. J. Hoogendijk W. J. Tilders F. J. (1995). Corticotropin-releasing hormone mRNA levels in the paraventricular nucleus of patients with Alzheimer’s disease and depression. *Am. J. Psychiatry* 152 1372–1376. 10.1176/ajp.152.9.1372 7653697

[B108] Renault P. F. Hoofnagle J. H. Park Y. Mullen K. D. Peters M. Jones D. B. (1987). Psychiatric complications of long-term interferon alfa therapy. *Arch. Intern. Med.* 147 1577–1580. 10.1001/archinte.147.9.1577 3307672

[B109] Reul J. M. Holsboer F. (2002a). Corticotropin-releasing factor receptors 1 and 2 in anxiety and depression. *Curr. Opin. Pharmacol.* 2 23–33. 10.1016/s1471-4892(01)00117-5 11786305

[B110] Reul J. M. Holsboer F. (2002b). On the role of corticotropin-releasing hormone receptors in anxiety and depression. *Dialogues Clin. Neurosci.* 4 31–46. 22033745 10.31887/DCNS.2002.4.1/jreul PMC3181666

[B111] Samuels B. A. Hen R. (2011). Neurogenesis and affective disorders. *Eur. J. Neurosci.* 33 1152–1159. 10.1111/j.1460-9568.2011.07614.x 21395859

[B112] Schwandt M. L. Cortes C. R. Kwako L. E. George D. T. Momenan R. Sinha R. (2016). The CRF1 antagonist verucerfont in anxious alcohol-dependent women: translation of neuroendocrine, but not of anti-craving effects. *Neuropsychopharmacology* 41 2818–2829. 10.1038/npp.2016.61 27109623 PMC5061889

[B113] Singhal G. Jaehne E. J. Corrigan F. Toben C. Baune B. T. (2014). Inflammasomes in neuroinflammation and changes in brain function: a focused review. *Front. Neurosci.* 8:315 10.3389/fnins.2014.00315 PMC4188030 25339862

[B114] Slominski A. T. Zmijewski M. A. Zbytek B. Tobin D. J. Theoharides T. C. (2013). Key role of CRF in the skin stress response system. *Endocr. Rev.* 34 827–84. 10.1210/er.2012-1092 23939821 PMC3857130

[B115] Smith E. M. Cadet P. Stefano G. B. Opp M. R. Hughes T. K. (1999). IL-10 as a mediator in the HPA axis and brain. *J. Neuroimmunol.* 100 140–148. 10.1016/S0165-5728(99)00206-4 10695724

[B116] Song C. Zhang X. Y. Manku M. (2009). Increased phospholipase A2 activity and inflammatory response but decreased nerve growth factor expression in the olfactory bulbectomized rat model of depression: effects of chronic ethyl-eicosapentaenoate treatment. *J. Neurosci.* 29 14–22. 10.1523/JNEUROSCI.3569-08.2009 19129380 PMC6664916

[B117] Spierling S. R. Zorrilla E. P. (2017). Don’t stress about CRF: assessing the translational failures of CRF1antagonists. *Psychopharmacology* 234 1467–1481. 10.1007/s00213-017-4556-2 28265716 PMC5420464

[B118] Steffens D. C. Helms M. J. Krishnan K. R. Burke G. L. (1999). Cerebrovascular disease and depression symptoms in the cardiovascular health study. *Stroke* 30 2159–2166. 10.1161/01.str.30.10.2159 10512922

[B119] Stetler C. Miller G. E. (2011). Depression and hypothalamic-pituitary-adrenal activation: a quantitative summary of four decades of research. *Psychosom. Med.* 73 114–126. 10.1097/PSY.0b013e31820ad12b 21257974

[B120] Stevens S. L. Shaw T. E. Dykhuizen E. Lessov N. S. Hill J. K. Wurst W. (2003). Reduced cerebral injury in CRH-R1 deficient mice after focal ischemia: a potential link to microglia and atrocytes that express CRH-R1. *J. Cereb. Blood Flow Metab.* 23 1151–1159. 10.1097/01.Wcb.0000086957.72078.D4 14526225

[B121] Sun X. Zhu F. Zhou J. Chang X. Li L. Hu H. (2018). Anti-migraine and anti-depression activities of tianshu capsule by mediating monoamine oxidase. *Biomed. Pharmacother.* 100 275–281. 10.1016/j.biopha.2018.01.171 29438841

[B122] Swaab D. F. Bao A. M. Lucassen P. J. (2005). The stress system in the human brain in depression and neurodegeneration. *Ageing Res. Rev.* 4 141–94. 10.1016/j.arr.2005.03.003 15996533

[B123] Taguchi R. Shikata K. Furuya Y. Ino M. Shin K. Shibata H. (2016). Selective corticotropin-releasing factor 1 receptor antagonist E2508 has potent antidepressant-like and anxiolytic-like properties in rodent models. *Behav. Brain Res.* 312 138–147. 10.1016/j.bbr.2016.06.017 27297028

[B124] Todorovic C. Sherrin T. Pitts M. Hippel C. Rayner M. (2009). Suppression of the MEK/ERK signaling pathway reverses depression-like behaviors of CRF2-deficient mice. *Neuropsychopharmacology* 34 1416–1426. 10.1038/npp.2008.178 18843268 PMC2680273

[B125] Utsuyama M. Hirokawa K. (2002). Differential expression of various cytokine receptors in the brain after stimulation with LPS in young and old mice. *Exp. Gerontol.* 37 411–420. 10.1016/S0531-5565(01)00208-X 11772528

[B126] Vale W. Spiess J. Rivier C. Rivier J. (1981). Characterization of a 41-residue ovine hypothalamic peptide that stimulates secretion of corticotropin and beta-endorphin. *Science* 213 1394–1397. 10.1126/science.6267699. 6267699

[B127] Vilhardt F. (2005). Microglia: phagocyte and glia cell. *Int. J. Biochem. Cell Biol.* 37 17–21. 10.1016/j.biocel.2004.06.010 15381143

[B128] Vreeburg S. A. Hoogendijk W. J. van Pelt J. Derijk R. H. Verhagen J. C. van Dyck R. (2009). Major depressive disorder and hypothalamic-pituitary-adrenal axis activity: results from a large cohort study. *Arch. Gen. Psychiatry* 66 617–626. 10.1001/archgenpsychiatry.2009.50 19487626

[B129] Wang W. Ji P. Dow K. E. (2003). Corticotropin-releasing hormone induces proliferation and TNF-alpha release in cultured rat microglia via MAP kinase signalling pathways. *J. Neurochem.* 84 189–195. 10.1046/j.1471-4159.2003.01544.x 12485415

[B130] Wang W. Shi W. Qian H. Deng X. Wang T. Li W. (2017). Stellate ganglion block attenuates chronic stress induced depression in rats. *PLoS One* 12:e0183995. 10.1371/journal.pone.0183995 28859148 PMC5578491

[B131] Wang Z. Zhang D. Hui S. Zhang Y. Hu S. (2013). Effect of tribulus terrestris saponins on behavior and neuroendocrine in chronic mild stress depression rats. *J. Tradit. Chin. Med.* 33 228–232. 10.1016/s0254-6272(13)60130-2 23789222

[B132] Waters R. P. Rivalan M. Bangasser D. A. Deussing J. M. Ising M. Wood S. K. (2015). Evidence for the role of corticotropin-releasing factor in major depressive disorder. *Neurosci. Biobehav. Rev.* 58 63–78. 10.1016/j.neubiorev.2015.07.011 26271720 PMC4828243

[B133] Weathington J. M. Hamki A. Cooke B. M. (2014). Sex- and region-specific pubertal maturation of the corticotropin-releasing factor receptor system in the rat. *J. Comp. Neurol.* 522(6), 1284–1298. 10.1002/cne.23475 24115088

[B134] Wiemelt A. P. Lehtinen M. McMorris F. A. (2001). Agonists calcitonin, corticotropin-releasing hormone, and vasoactive intestinal peptide, but not prostaglandins or beta-adrenergic agonists, elevate cyclic adenosine monophosphate levels in oligodendroglial cells. *J. Neurosci. Res.* 65 165–172. 10.1002/jnr.1139 11438985

[B135] Wohleb E. S. Franklin T. Iwata M. Duman R. S. (2016). Integrating neuroimmune systems in the neurobiology of depression. *Nat. Rev. Neurosci.* 17 497–511. 10.1038/nrn.2016.69 27277867

[B136] Xu F. Peng D. Tao C. Yin D. Kou J. Zhu D. (2011). Anti-depression effects of danggui-shaoyao-san, a fixed combination of traditional chinese medicine, on depression model in mice and rats. *Phytomedicine* 18 1130–1136. 10.1016/j.phymed.2011.05.002 21664113

[B137] Yang S. J. Yu H. Y. Kang D. Y. Ma Z. Q. Qu R. Fu Q. (2014). Antidepressant-like effects of salidroside on olfactory bulbectomy-induced pro-inflammatory cytokine production and hyperactivity of HPA axis in rats. *Pharmacol. Biochem. Behav.* 124 451–457. 10.1016/j.pbb.2014.07.015 25101546

[B138] You T. T. Cheng Y. F. Zhong J. H. Bi B. T. Zeng B. Q. Zheng W. H. (2017). Roflupram, a phosphodiesterase 4 inhibitior, suppresses inflammasome activation through autophagy in microglial cells. *ACS Chem. Neurosci.* 8 2381–2392. 10.1021/acschemneuro.7b0005 28605578

[B139] Yue N. Huang H. Zhu X. Han Q. Wang Y. Li B. (2017). Activation of P2X7 receptor and NLRP3 inflammasome assembly in hippocampal glial cells mediates chronic stress-induced depressive-like behaviors. *J. Neuroinflamm.* 14:102. 10.1186/s12974-017-0865-y 28486969 PMC5424302

[B140] Zhu W. Zheng H. H. Shao X. L. Wang W. Yao Q. Li Z. L. (2010). Excitotoxicity of TNF alpha derived from KA activated microglia on hippocampal neurons in vitro and in vivo. *J. Neurochem.* 114 386–396. 10.1111/j.1471-4159.2010.06763.x 20438614

[B141] Zhu Y. Duan X. Cheng X. Cheng X. Li X. Zhang L. (2016). Kai-Xin-San, a standardized traditional chinese medicine formula, up-regulates the expressions of synaptic proteins on hippocampus of chronic mild stress induced depressive rats and primary cultured rat hippocampal neuron. *J. Ethnopharmacol.* 193 423–432. 10.1016/j.jep.2016.09.037 27660009

[B142] Zoumakis E. Rice K. C. Gold P. W. Chrousos G. P. (2006). Potential uses of corticotropin-releasing hormone antagonists. *Ann. N. Y. Acad. Sci.* 1083 239–251. 10.1196/annals.1367.021 17148743

